# Physiological Concentration of Exogenous Lactate Reduces Antimycin A Triggered Oxidative Stress in Intestinal Epithelial Cell Line IPEC-1 and IPEC-J2 *In Vitro*

**DOI:** 10.1371/journal.pone.0153135

**Published:** 2016-04-07

**Authors:** Stefan Kahlert, Sami Junnikkala, Lydia Renner, Ulla Hynönen, Roland Hartig, Constanze Nossol, Anikó Barta-Böszörményi, Sven Dänicke, Wolfgang-Bernhard Souffrant, Airi Palva, Hermann-Josef Rothkötter, Jeannette Kluess

**Affiliations:** 1 Institute of Anatomy, Otto von Guericke University Magdeburg, Magdeburg, Germany; 2 Institute of Animal Nutrition, Friedrich-Loeffler-Institute, Federal Research Institute for Animal Health, Braunschweig, Germany; 3 Institute of Molecular and Clinical Immunology, Otto von Guericke University Magdeburg, Magdeburg, Germany; 4 Department of Veterinary Biosciences, University of Helsinki, Helsinki, Finland; 5 Research Unit for Nutritional Physiology ‘Oskar Kellner’, Leibniz Institute for Farm Animal Biology, Dummerstorf, Germany; University of Chinese Academy of Sciences, CHINA

## Abstract

Weaning triggers an adaptation of the gut function including luminal lactate generation by lactobacilli, depending on gastrointestinal site. We hypothesized that both lactobacilli and lactate influence porcine intestinal epithelial cells. *In vivo* experiments showed that concentration of lactate was significantly higher in gastric, duodenal and jejunal chyme of suckling piglets compared to their weaned counterparts. In an *in vitro* study we investigated the impact of physiological lactate concentration as derived from the *in vivo* study on the porcine intestinal epithelial cells IPEC-1 and IPEC-J2. We detected direct adherence of lactobacilli on the apical epithelial surface and a modulated F-actin structure. Application of lactobacilli culture supernatant alone or lactate (25 mM) at low pH (pH 4) changed the F-actin structure in a similar manner. Treatment of IPEC cultures with lactate at near neutral pH resulted in a significantly reduced superoxide-generation in Antimycin A-challenged cells. This protective effect was nearly completely reversed by inhibition of cellular lactate uptake via monocarboxylate transporter. Lactate treatment enhanced NADH autofluorescence ratio (F_cytosol_/F_nucleus_) in non-challenged cells, indicating an increased availability of reduced nucleotides, but did not change the overall ATP content of the cells. Lactobacilli-derived physiological lactate concentration in intestine is relevant for alleviation of redox stress in intestinal epithelial cells.

## Introduction

The intestinal epithelium is the largest border between the body and the environment. In contrast to the skin, the mission of the intestinal epithelium, namely processing and transport of all kinds of nutrient matter, involves an intensive interaction with the environment. Therefore the enterocyte is one of the first targets of various kinds of harmful factors, e.g. enteric pathogens or toxins [1, 2].

The functions of enterocytes are energy-consuming processes, which necessitate oxygen-based respiration. The reduction of oxygen involves the handling of highly reactive oxygen species, which can be highly toxic if not carefully controlled [3]. The mitochondrial respiration is one of the major sources of reactive oxygen species (ROS), namely superoxide, in respirating cells and a cascade of reactions is dedicated for the detoxification of this side product of oxidative phosphorylation [4]. The generation of ROS is detectable in living cells using ROS sensitive fluorescence dyes which allow the detection of different ROS species [5, 6]. Moreover, detoxification mechanisms are dependent on the availability of reduction equivalents, a parameter which can be followed detecting NADH autofluorescence [7].

Lactobacilli are commensal bacteria and it has been shown that their presence and activity is beneficial for gut health [8]. A large array of molecular interactions between so called probiotics is described emphasizing the relevance of the microbiota for the physiology and immune function of the gut [9]. The protection of epithelial tight junctions by probiotics has been shown in a hydrogen peroxide challenge model *in vitro* [10].

*L*. *amylovorus* was selected because it is characteristic for the pig’s intestine. The strains used here were isolated from pig and it has been shown that the numbers of lactobacilli, especially those of *L*. *amylovorus*, decrease during weaning [11, 12].

The first massive change in intestinal physiology in the life of mammals is the switch from mother milk to solid feed. During this period the intestinal microbiota adapts to the feed of the adult mammal. It has been shown that during this time the oxidative stress is enhanced [13].

Here, we have analysed the effect of lactate as a principal lactobacillus metabolite on the enterocyte cell physiology, especially on the ROS handling and redox status after a challenge *in vitro*. Lactate concentrations measured *in vivo* in chyme samples were applied to mimic the *in vivo* situation *in vitro* on porcine cell lines IPEC-1 and IPEC-J2.

## Materials and Methods

### Animal Experiment

The experiment was performed in the swine facilities at the Leibniz Institute for Farm Animal Biology, Nutritional Physiology “Oskar Kellner” in Dummerstorf, Germany and was approved by the ethical committee of the Ministry for Nutrition, Agriculture, Forestry and Fishery Mecklenburg-Vorpommern. The trial has been described in detail before [14].

Briefly, piglets of both genders (German Landrace) were raised conventionally from the sow in farrowing pens with sow’s milk only and no access to creep feed. At 28 d of life piglets were weaned, re-allocated into group pens each containing 8 animals, and provided with a starter diet (meal) *ad libitum*. Diets were formulated to meet requirements of weaning piglets [15]. Animals were sacrificed before and after weaning by intracardial injection of 2 mL T61^R^ (Intervet, Germany), and digesta was collected from stomach, small intestine, caecum and colon. The small intestine was divided into three equal sections in proximo-distal direction and designated as duodenum, jejunum and ileum. Chyme of 4 animals was pooled to obtain sufficient material and thus we obtained preweaning 8 and postweaning 40 pool samples. Those samples were processed for analysis of lactate and *Lactobacillus spp*. counts and pH was measured immediately after sampling.

Total lactate was measured by colorimetric procedure [16] and *Lactobacillus spp*. was cultivated on MRS agar plates (SIFIN®, Berlin, Germany) at 37°C in anaerobic jars (Anaerocult® A, Merck, Germany) for 72 h. Colony-forming units (cfu) were counted and numbers are presented as log cfu/g digesta.

### IPEC-1 and IPEC-J2 Cell Culture Conditions

IPEC-1 (ACC705) cell line was used in this study as previously described [17]. Briefly, cells were cultured in Dulbecco’s modified eagle medium (DMEM/Ham’s F-12 (1:1)) supplemented with 5% fetal calf serum (FCS), 1% insulin-transferrin-selenium (ITS), 16 mmol/L HEPES (all PAN-Biotech) and 5 ng/mL epidermal growth factor (EGF; BD Biosciences), incubated at 39°C and 5% CO_2_. Cell cultures were regularly tested and found to be free of mycoplasma contamination (Venor^®^ GeM Mycoplasma Detection Kit; Minerva Biolabs). Cells were routinely seeded at a density of 0.6 x 10^5^/mL (IPEC-1) with 7.5 mL medium in plastic tissue culture flasks (25 cm^2^ Nunc) and passaged every 3–4 d for a maximum of 20 times (IPEC-1 passages 103-123). Additionally, ROS measurements were performed in parallel with IPEC-J2 (ACC701) cells [18]. IPEC-J2 cell line was cultured as described for IPEC-1 except a lower seeding density of 0.5 x 10^5^/mL. IPEC-J2 passages were used between 78 and 98. For all microscopic based methods cells were seeded on uncoated 35 mm glass bottom dishes (1.5 cm^2^ glass surface, MatTek) where they formed a confluent monolayer within 7 d (0.2 x 10^6^ cells/cm^2^) and were subsequently used in the experiments.

### *Lactobacillus* Culture, Bacterial Adhesion and Supernatant Preparation

Bacteria from glycerol stocks of three strains of *Lactobacillus amylovorus* (GRL1110 = DSM16698; GRL1112; GRL1115) [19] were cultured in 10 mL MRS-broth (BD Difco™) each in anaerobic jars (Anaerocult^®^ A, Merck Millipore) at 39°C for 16–18 h. Bacteria were centrifuged at 5000 x g for 10 min and re-suspended in DMEM (FCS-free) media. Direct bacteria-IPEC interaction was monitored by application of 2 x 10^9^ bacteria on a confluent IPEC-1 cell layer resulting in a cell:bacteria ratio of 1:1000. After 6 h bacterial suspension was carefully removed and cultures were fixed by 4% paraformaldehyde (PFA) or ethanol / acetone according to subsequent staining (see below). Bacteria and enterocyte nuclei were visualized by DAPI staining. The effect of soluble components of lactobacilli was analysed by application of bacterial culture supernatants on IPEC-1 cells. Supernatants were obtained by inoculating DMEM/Ham’s F-12 (1:1) medium without FCS with 1 x 10^9^ bacteria / mL and cultivating for 6 h at 39°C. Afterwards the suspension was centrifuged at 2000 x g for 10 min and the supernatant was filtrated through a 0.22 μm syringe filter. The pH values of supernatants were found to vary between 4.0 and 4.2. Supernatants (2 mL) were applied for 1 h (39°C) on confluent IPEC-1 cells grown on glass bottom dishes. After removal of the supernatants the cells were fixed with PFA as described below.

### Epifluorescence and Confocal Microscopy

IPEC-1 cells and attached bacteria were fixed with 4% PFA for 10 min at RT, washed 3 times with 0.1 M phosphate buffered saline (PBS) without Ca^2+^ and Mg^2+^ and permeabilised with 1% Triton X-100 for 5 min. F-actin staining was performed by blocking with normal goat serum (NGS, 1:100) for 30 min (Axxora) followed by an incubation for 30 min with PromoFluor546-phalloidin (1:40, Promocell) at RT. Subsequently, glass bottom dishes were incubated with DAPI (1:10, Partec) for 5 min, washed with PBS and inspected using an inverted microscope (Zeiss Axiovert 200M, Plan-Neofluar 40x, 1.3). For DAPI staining alone, cells were washed three times with PBS and treated with ethanol (30 min, 4°C) and acetone (100 μL, -20°C, 90 s), stained with DAPI and inspected by an inverted microscope.

Visualisation of IPEC-1 plasma membrane was performed by loading of CellTracker CM-Dil (1 μM, Invitrogen) for 5 min. Cells and attached bacteria were fixed by 4% PFA, counterstained with DAPI and submersed with PBS. Three dimensional structures were analysed by confocal microscopy using a Leica SP2 confocal microscope (Leica) and orthogonal projection as well as 3D reconstruction of the optical sections (ImageJ, NIH).

Cryosections of porcine jejunum (5 μm) were fixed in methanol-acetone, permeabilised with 0.3% Triton X100 (Sigma-Aldrich) for 5 min and blocked with 10% NGS for 30 min. F-actin was detected by incubation with PromoFluor546-phalloidin (1:40, Promocell) at RT for 1 h and nuclei were counterstained with DAPI. Sections were embedded in mounting medium (Mowiol; Calbiochem).

### Generation of ROS *In Vitro*

**Fig 1:**
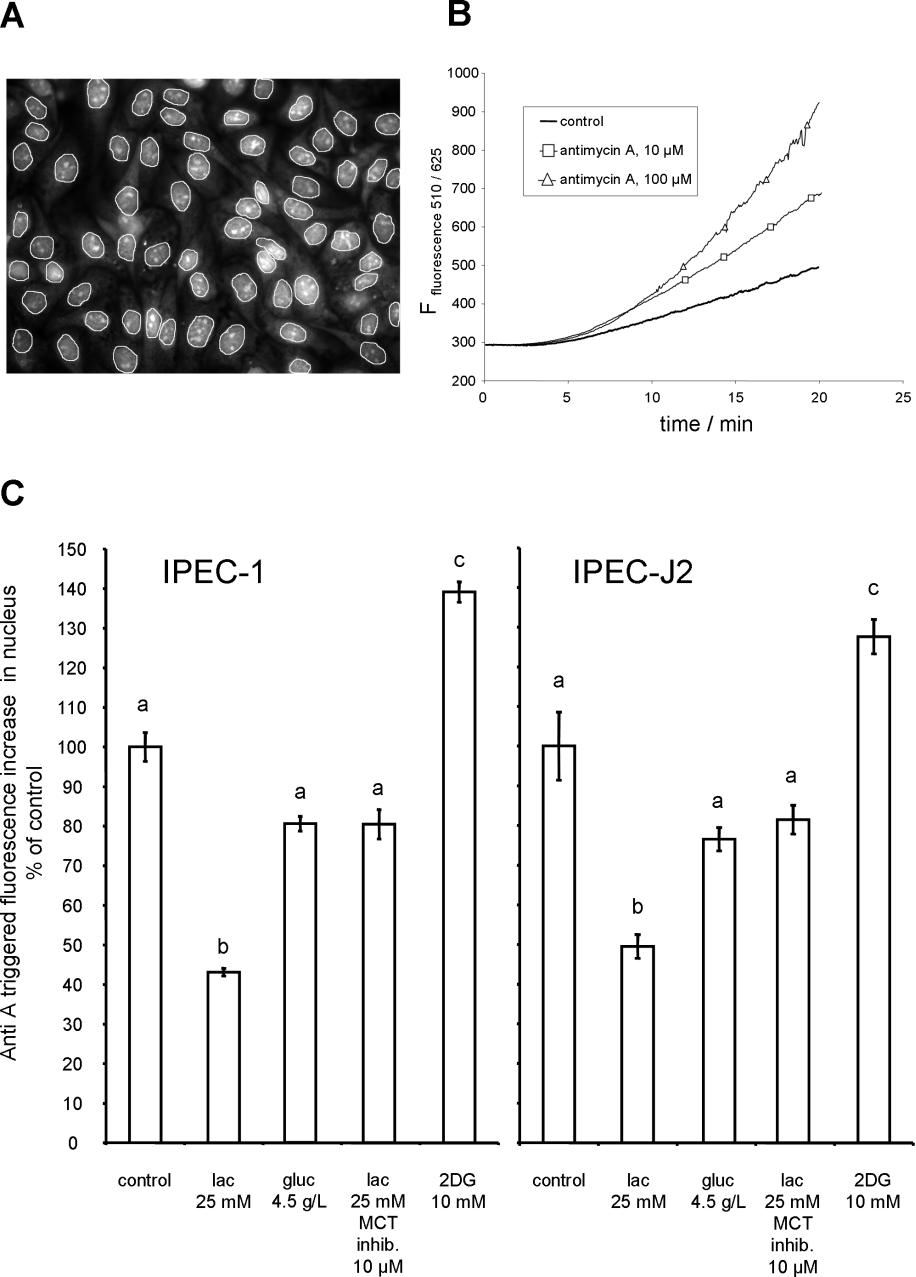
Normalised Anti A triggered ROS generation in IPEC cells after lactate and glucose treatment. **(A)** IPEC-1 cells were cultured on glass (7 d), incubated for 1 h in buffer (HBSS), placed on microscopic stage and challenged with Anti A in the presence of DHE. Mean fluorescence of nuclei (regions of interest, white circles) was monitored over time. **(B)** Quantification of nucleic fluorescence of ethidium cation (ethidium cation is the oxidation product of DHE) binding to nuclei. Anti A (10 / 100 μM) or control buffer was applied in presence of DHE 1.5 min after starting the measurement. **(C)** Anti A (100 μM) triggered superoxide generation rate (DHE oxidation) of IPEC-1 and IPEC-J2 cells after 1 h treatment (39°C). Cells were treated with lactate (lac, 25 mM); lac + inhibitor of monocarboxylate transporter (MCT inhibitor, ARC155858, 10 μM); increased glucose (gluc, 4.5 g/L) or glycolysis inhibitor 2-Deoxyglucose (2DG, 10 mM). Glucose concentration was 1 g/L if not otherwise indicated. Slope of fluorescence increase of individual cell nuclei was calculated and normalised to the control. Statistical analysis was done by Kruskal-Wallis and Dunn's post-hoc test. Different superscripts indicate statistically significant (p<0.05) differences.

Generation of reactive oxygen species was detected by oxidation of non-fluorescent dihydroethidium (DHE) to ethidium^+^. The subsequent intercalation of ethidium^+^ with DNA allows the fluorescence detection in the nucleus (see **Fig 1**). The nuclear fluorescence is a measure of predominantly superoxide generation [20]. IPEC-1 and IPEC-J2 were cultured on glass bottom dishes and treated with Hank´s balanced salt solution (HBSS), pH 7.3, supplemented with glucose (D-(+) glucose, Sigma-Aldrich G7021, 1g/L or 4.5 g/L), glucose (1g/L) + lactate (DL-lactate, sodium salt, Sigma-Aldrich L4263, 25 mM), glucose (1 g/L) + lactate (25 mM) + AR-C 155858 (Haoyuan Chemexpress HY-13248, 10 μM, MCT1+2 inhibitor [21]) or glucose (1 g/L) + 2-Deoxyglucose (2DG, Sigma-Aldrich D6134, 10 mM) for 60 min at 39°C. After 60 min incubation cells were placed on a tempered microscopic stage (AxioVert, Zeiss, equipped with a monochromator (TILL vision) and a video camera (Exif Aqua)) and challenged with Antimycin A (Anti A, Sigma-Aldrich A8674, 100 μM) in the presence of DHE (Invitrogen, D11347, 10μM) and the corresponding treatment solution. Kinetic of fluorescence increase (excitation 510 nm, emission 625 nm) was recorded in 3 s intervals (50 ms illumination time) for up to 20 min. Fluorescence slope of nuclei between 10 min and 15 min was analysed using Fiji software (NIH). Single cell measurements of different cell lines and treatments were repeated at least 3 times monitoring 20–50 (IPEC-1) or 10–20 (IPEC-J2) cells each.

### Detection of Cytosolic Autofluorescence

**Fig 2:**
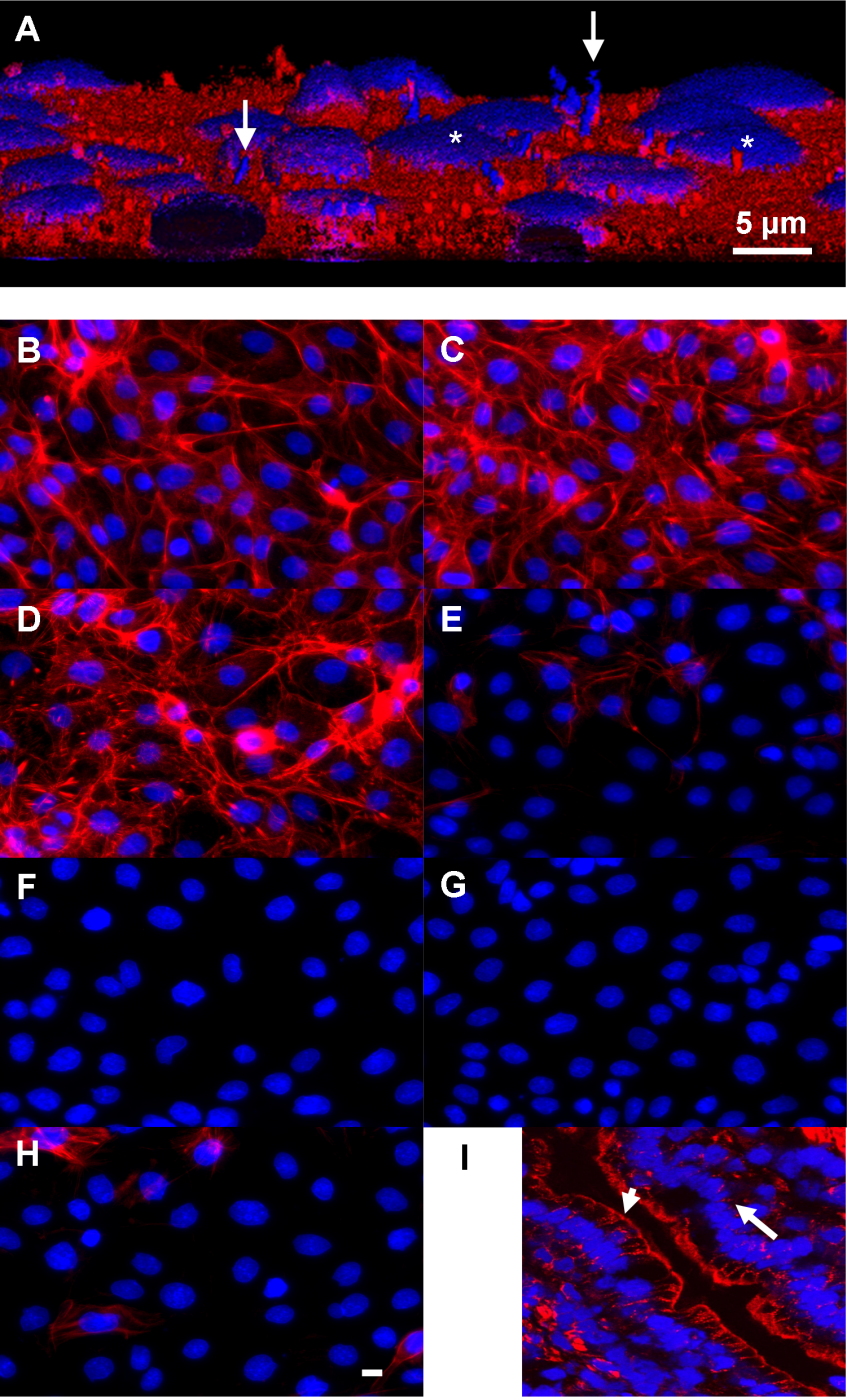
Attachment and structural effects of lactobacilli on IPEC-1 cells. **(A)** Three dimensional reconstruction of lactobacilli attached to IPEC-1 cells. *Lactobacillus amlylovorus* strain GRL1110 (arrows) was applied for 6 h on confluent IPEC-1 cells. The bacterial suspension was then carefully removed; cells and attached bacteria were fixed and stained with DAPI (nuclei, blue) and CM-Dil (plasma membrane, red). Confocal sections were reconstructed to obtain a 3D view. Note the thin, barely visible cytosolic layer above nucleus (*). (**B-H**) Actin structure of IPEC-1 cells treated with lactate and *Lactobacillus amylovorus* supernatants. IPEC-1 cells were cultured on glass, incubated as indicated and stained for F-actin (Phalloidin-PromoFluor 546) and nuclei (DAPI). (**B**) control buffer pH7, (**C**) Na-DL-lactate (25 mM, pH 7), (**D**) control buffer pH 4, (**E**) lactic acid pH 4. (**F-H**) supernatants of *Lactobacillus amylovorus* strains. (**F**) GRL1110, (**G**) GRL1112, (**H**) GRL1115. Predominantly basal F-actin fibre structure was reduced by lactic acid and bacterial supernatants (pH 4), (**I**) F-actin staining of freeze-section of porcine jejunal epithel. Note positive F-actin signal indicating the apical terminal web (arrowhead), the low F-actin signal at the lateral side and the faint signal in the basal region of the enterocytes (arrow). Bar: 5 μm.

**Fig 3:**
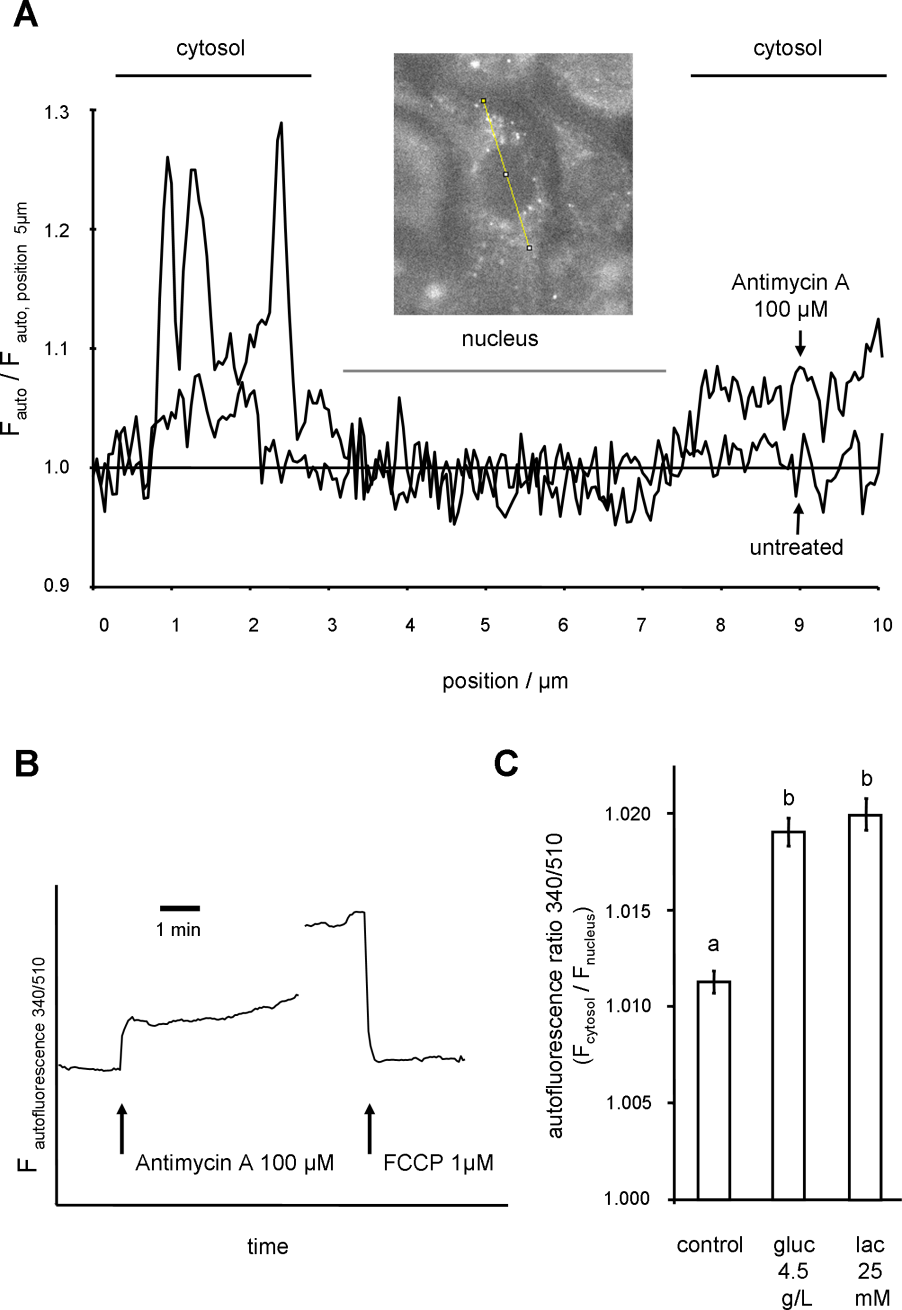
Quantification of cytosol / nucleus NADH ratio in IPEC-1 cells after 1 h treatment with glucose and lactate without further challenge. Cellular autofluorescence of IPEC-1 cells was monitored at ex: 340 nm / em: 510 nm. **(A)** Change in spatial distribution of autofluorescence in response to Anti A (100 μM). **(B)** Cytosolic autofluorescence in response to complex III inhibitor Anti A (100 μM) and uncoupler FCCP (1 μM). **(C)** IPEC-1 cells were treated as indicated for 1 h. Glucose (gluc) concentration was 1 g/L if not otherwise indicated. Cells were treated with lactate (lac) 25 mM or increased gluc 4.5 g/L. Autofluorescence measured after 1 h and ratio of cytosolic (F_cytosol_) and nuclei (F_nucleus_) was calculated (ex: 340 nm / em: 510 nm) from individual cells without further stimulation. Statistical analysis was done by Kruskal-Wallis and Dunn's post-hoc test. Different superscripts indicate statistically significant (p<0.05) differences.

The cellular autofluorescence signal is largely dependent on the mitochondrial NADH content [7]. In cultured cells, the majority of mitochondria is found in the cytosol surrounding the nucleus, whereas only a small proportion of mitochondria is found in the cytosolic layer above the nucleus [22]. In the cell culture used this layer is barely visible as shown in **Fig 2**. However, due to the limited optical resolution, a separation between cytosolic and mitochondrial NADH signal in the peri-nuclear area was not possible. IPEC-1 cells were cultured on glass bottom dishes as described above and treated with HBSS, pH 7.3 supplemented with glucose (1 g/L or 4.5 g/L), glucose (1 g/L) + lactate (25 mM), or glucose (1g/L) + 2DG (10 mM) for 60 min at 39°C. Dishes were transferred to the microscopic stage as described above and autofluorescence (excitation: 340 nm, emission: 510 nm) was monitored (3 s intervals) for up to 1.5 min without further manipulation. The fluorescence signal was stable during this time window and fluorescence images obtained after 60 s were further analysed as shown in **Fig 3**. FCCP (Sigma-Aldrich, C2929, 1 μM) was used as uncoupler. Due to the small overall effect measurements were repeated at least 5 times monitoring 20 to 50 cells each.

### Cellular ATP Level in IPEC-1 Cells

The effect of lactate on cellular ATP level was quantified using a commercial ATP kit (ATP Bioluminescence Assay Kit HS II, Roche). Briefly, IPEC-1 cells were seeded (0.6 x 10^5^/mL) in 96-well cell culture plates and cultured for 7 d. 6 wells were incubated for 60 min in parallel for each treatment (100 μL/well) as indicated. Supernatant was completely removed and cell layer was lysed by "Cell Lysis Reagent" (50 μL) for 5 min. The lysate of the 6 corresponding wells was pooled and ATP content was analysed by recording the luminescence kinetic after automatic injection of "Luciferase reagent". ATP content is given as percentage of untreated control of 5 independent experiments.

### Statistical Methods

Statistical analysis of the animal experiment was performed with the procedure “MIXED” (SAS, version 8) and comparison between pre- and postweaning using the t-test (p<0.01). Analysis of *in vitro* experiments was done by Kruskal-Wallis and Dunn’s post-hoc test.

## Results

### Luminal Lactic Acid Concentrations along the Intestinal Axis

**Fig 4:**
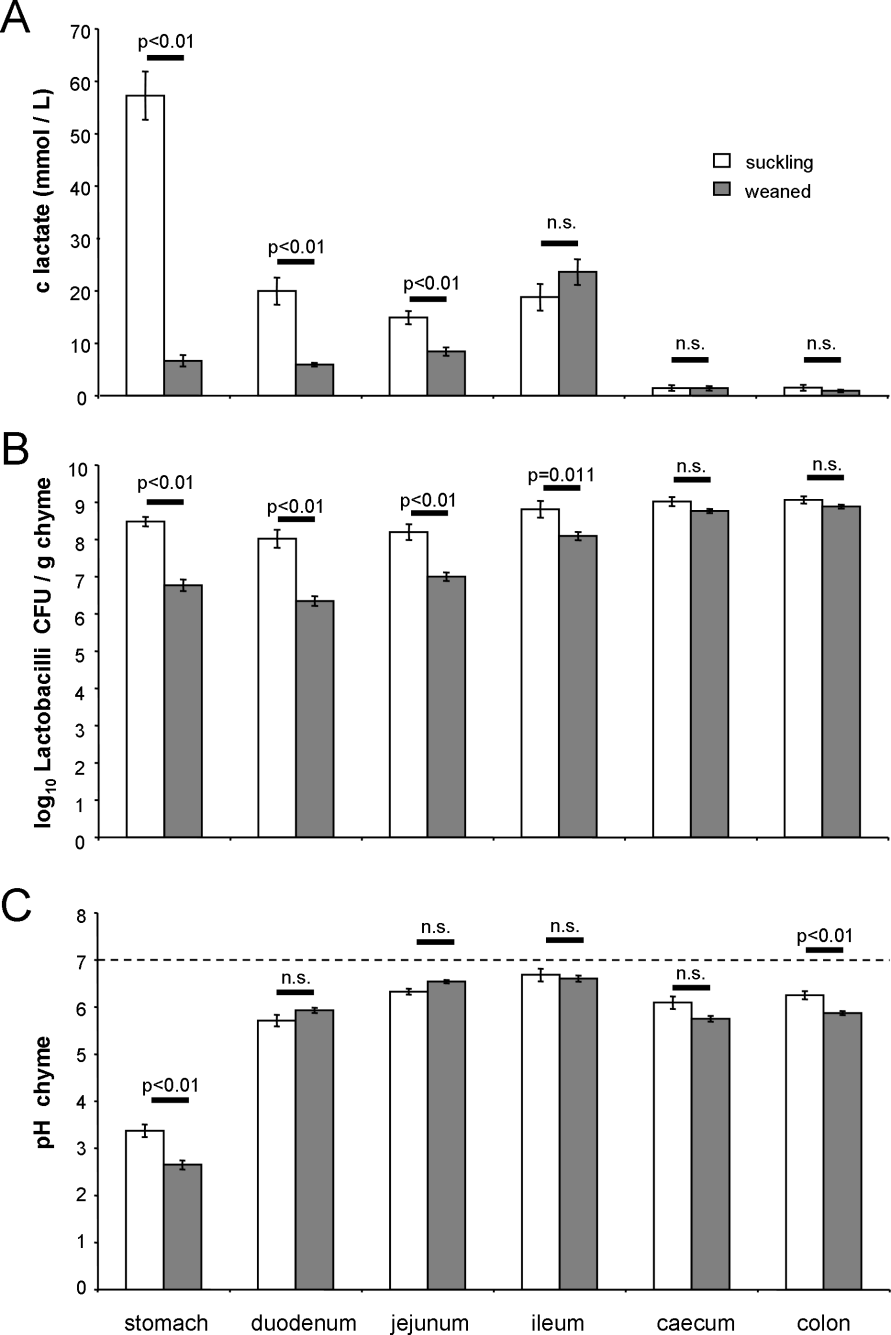
Effect of weaning on intestinal lactate concentration and lactobacilli load of piglets. **(A)** Lactate concentration **(B)** *Lactobacillus* load and **(C)** pH in chyme of suckling and weaned piglets along the intestinal axis was measured. Statistical analysis was performed with the procedure “MIXED” (SAS, version 8) and comparison between pre- and postweaning using the t-test (p<0.01).

In **Fig 4A** the lactic acid concentration of intestinal content of piglets is shown as measured in suckling and weaned piglets. As expected by the intake of maternal milk and fermentation in stomach [23] a high lactic acid load in the proximal sections of the intestinal tract was found together with a decrease to distal sections (stomach > duodenum = jejunum = ileum > caecum- = colon). Weaning led to a massive significant decrease of lactate in stomach, duodenum and jejunum, whereas the lactate concentration in ileum, caecum and colon was not significantly different. The lowest numerical values were recorded in caecum and colon (1-2 mmol/L) independent of feeding. In parallel the amount of lactobacilli in the intestine was recorded (**Fig 4B**). The *Lactobacillus* load of stomach, duodenum and jejunum was significantly reduced in response to weaning, but not in ileum, caecum and colon. In caecum and colon high *Lactobacillus* loads were found, however, the lactate concentration was low. The pH value of chyme (**Fig 4C**) decreased only in stomach and colon significantly in weaning piglets. Luminal pH in small intestinal sections (duodenum, jejunum and ileum) and caecum were not influenced by weaning. Jejunal and ileal chyme pH was in the neutral or weak acidic range (pH 6.3–6.7), irrespective of suckling or weaning.

Next, we analysed functional consequences of such lactate concentrations in neutral pH on enterocytes and utilized intestinal porcine cell lines IPEC-1 and IPEC-J2. Both cell lines are of small intestine origin and can therefore be used to mimic the *in vivo* situation in pig [18]. In consequence of the *in vivo* studies we chose a lactic acid concentration of 25 mmol/L as a realistic level for further *in vitro* studies. Due to the adherence of lactobacilli on enterocyte surface (shown below and [19]) the local lactate concentration is likely higher than measured in chyme.

### Adherence of Lactobacilli to IPEC-1 Cells *In Vitro* and Influence of Lactic Acid on the Morphology of the Cell Culture Model

**Fig 5:**
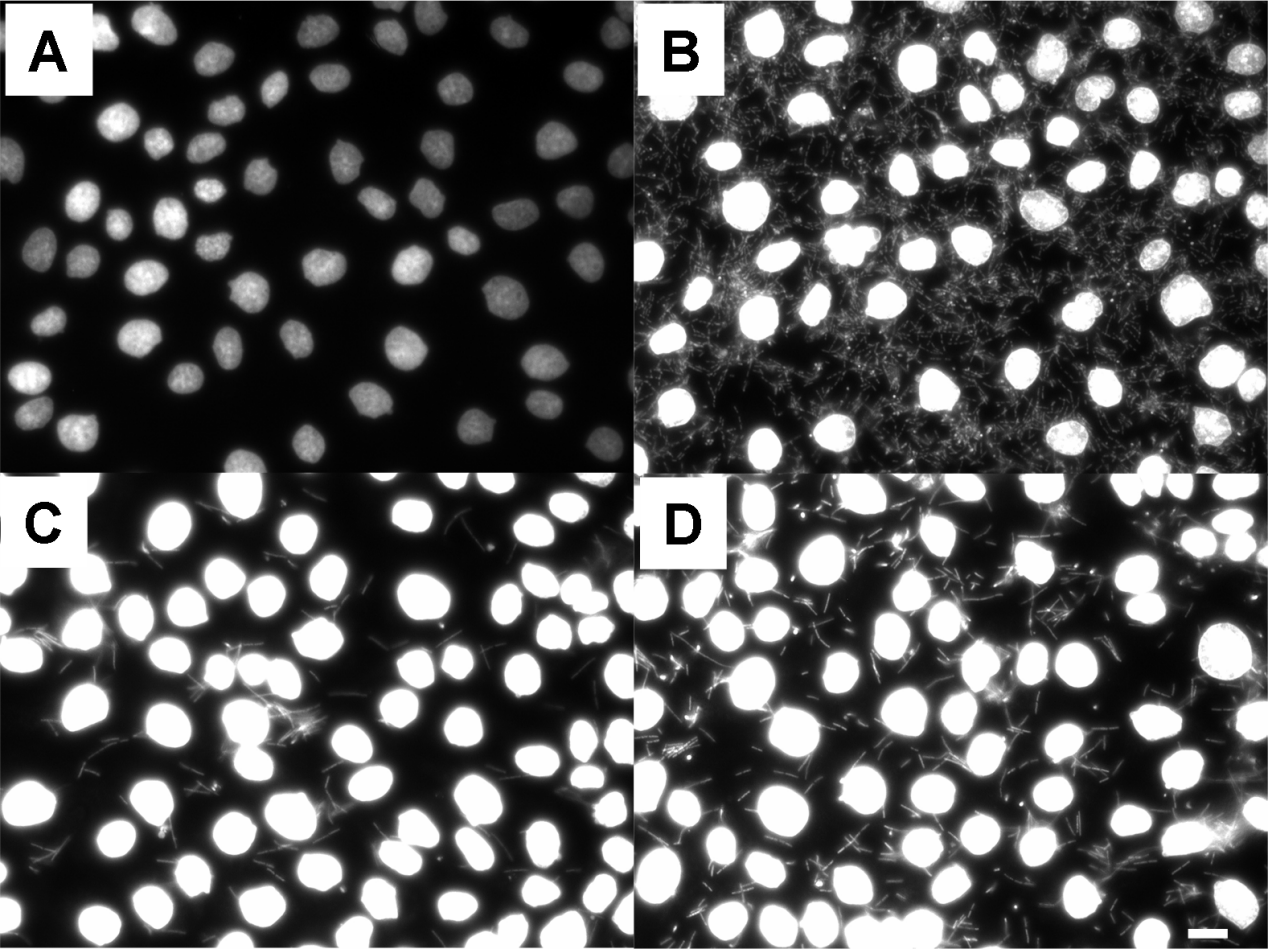
Visualisation of adherence of bacteria on IPEC-1 cells. Three different strains of *Lactobacillus amylovorus* (GRL1110 (**B**); GRL1112 (**C**); GRL1115 (**D**)) were applied on confluent IPEC-1 cells at the cell:bacteria ratio 1:1000 for 6 h in cell culture medium. Bacterial suspensions were removed, cells and attached bacteria were fixed and stained with DAPI. Bacterial DNA indicates a different degree of adherence of bacteria to epithelial cell surface (**B-D**) in comparison to non-treated (**A**) cells. Representative micrographs of three independent experiments are shown. Bar: 5 μm.

In the first step analysing the effect of lactobacilli on IPEC-1 cells we added *Lactobacillus amylovorus* on confluent IPEC-1 enterocyte cell line. Attached bacteria were stained with DAPI and are visible as rod-shaped bacteria (**Fig 5**). Differences in the degree of *Lactobacillus* attachment to the cell layer are visible however; all strains adhered under the selected conditions. Microscopic inspection of the IPEC cultures did not indicate any sign of cell loss or induction of apoptosis (e.g. nucleus fragmentation). 3D reconstruction of confocal immunofluorescence sections shows the *Lactobacillus* strain GRL1110 vertically attached on the surface of the IPEC-1 cell layer (**Fig 2A**). We found that lactobacilli can adhere to enterocyte surface *in vitro* as expected. In **Fig 2B–2H**the effect of lactic acid and supernatants of *Lactobacillus amylovorus* on actin cytoskeleton structure of glass-bottom cultured IPEC-1 cells is shown. In a previous experiment we found a modulation of actin structure after application of living bacteria (results not shown). In (**B**) typical basal stress fibres are visible when cells were cultured for 7 d and then treated at neutral pH for 1 h. The structure is not changed by the supplementation of 25 mmol/L lactate at neutral pH (**C**) or a shift acidic pH (pH 4) alone (**D**). The combined application of low pH and 25 mmol/L lactate drastically reduced the structure and amount of Phalloidin-detectable fibres (**E**). Neither the pH shift nor the lactate application at neutral pH alone had a comparable effect. In contrast, the differences in actin structure are also found with culture supernatants (pH 4 in supernatant after 16–18 h of cultivation) of three different lactobacilli strains (**F-H**). The morphology of the IPEC-1 cell layer is not changed as judged from bright field views. *In vivo* Phalloidin-detectable fibres are prominent at the luminal side (terminal web) of the enterocytes (**I**), however, a basal staining resembling the actin structures as seen *in vitro* (e.g. **B**) is very weak. The findings show that realistic lactate concentrations influence the structure of enterocytes *in vitro* only at low pH values below the pH measured *in vivo*.

### Influence of Lactate on the ROS Handling of Anti A Challenged IPECs

In the next step we analysed the role of lactate on the cellular handling of reactive oxygen species. To do this, we challenged the cell system by Anti A (100 μM). Due to blockade of the complex III of the respiration chain, superoxide radicals are generated. The amount of superoxide generation was measured by oxidation of DHE to ethidium cation. The oxidation of DHE is predominantly due to the superoxide radical [20]. The binding of ethidium^+^ to nuclear DNA results in a measurable fluorescence signal (**Fig 1A**). Anti A (100μM) challenge of IPEC-1 cells trigger a dose dependent increase in nucleic fluorescence due to superoxide leakage from respiratory chain (**Fig 1B**). The influence of lactate and glucose on the Anti A mediated epithelial superoxide generation was tested in IPEC-1 and IPEC-J2 cell line. As shown in **Fig 1C** the pre-treatment (60 min) and presence of lactate (25 mM) reduced the Anti A mediated superoxide generation significantly (below 50% of the control (HBSS)). The inhibition of lactic acid uptake by MCT inhibitor ARC155858 (10 μM) impaired the ROS reduction and the DHE oxidation rate was no longer significantly lower compared to the control. We further analysed the influence of glucose on superoxide generation. In control conditions the buffer (HBSS) contains 1 g/L glucose and the Anti A- triggered DHE oxidation rate is normalised to 100%. An enrichment of glucose (4.5 g/L) reduced the superoxide generation numerically in comparison to control. In contrast, inhibition of glycolysis by 2DG in the presence of 1 g/L glucose enhanced the superoxide generation rate significantly.

### Effect of Lactate on NADH Handling

As a possible mechanism underlying the protective effect of lactate against superoxide the available amount of NADH was analysed. Scavenging mechanisms are dependent on a reduction step fed by NADH of NAD(P)H. The fluorescence properties of NAD^+^/NADH allow the detection of NADH without introduction of sensitive fluorescence dyes into the cell [7]. Typically, mitochondrial-rich cytosolic regions exhibit a strong autofluorescence signal. As shown in **Fig 3A,** the autofluorescence signal of the mitochondria-rich cytosolic region increased when the respiratory chain was blocked with Anti A (100 μM). This effect can be explained by the accumulation of NADH due to the blockade of complex III of respiration chain. In line with this explanation the application of uncoupler (FCCP, 1 μM) decreased the NADH autofluorescence as the amount of mitochondrial NADH was reduced by maximal use of NADH by the respiratory chain (**Fig 3B**). The mitochondria are localised within the cytosol whereas the thin region above the nucleus and the nucleus itself is nearly or completely free of mitochondria and did not exhibit a relevant signal. Moreover, due to the thin cytosolic layer above the nucleus the NADH signal from the cytosol in this area was negligible. Therefore, the nuclei area was used as a reference allowing the normalisation of the autofluorescence level (**Fig 3A**). The obtained signal was designated as autofluorescence ratio F_cytosol_/F_nucleus_. In contrast to the fast and strong effects of inhibitors of mitochondrial respiration on the autofluorescence ratio, the effects of lactate and glucose are smaller and recorded after 60 min of incubation (**Fig 3C**). After 1 h of incubation the ratio of the control (HBSS buffer, 1 g/L glucose) is 1.012 and with lactate 1.018 (HBSS buffer, 1 g/L glucose + 25 mM lactate). Nevertheless, a significant increase in autofluorescence ratio is found with lactate or high glucose (4.5 g/L). Note that the autofluorescence ratios are obtained without application of fluorescence dyes or mitochondrial toxins.

### Effect of Lactate on Cellular ATP Level

**Fig 6:**
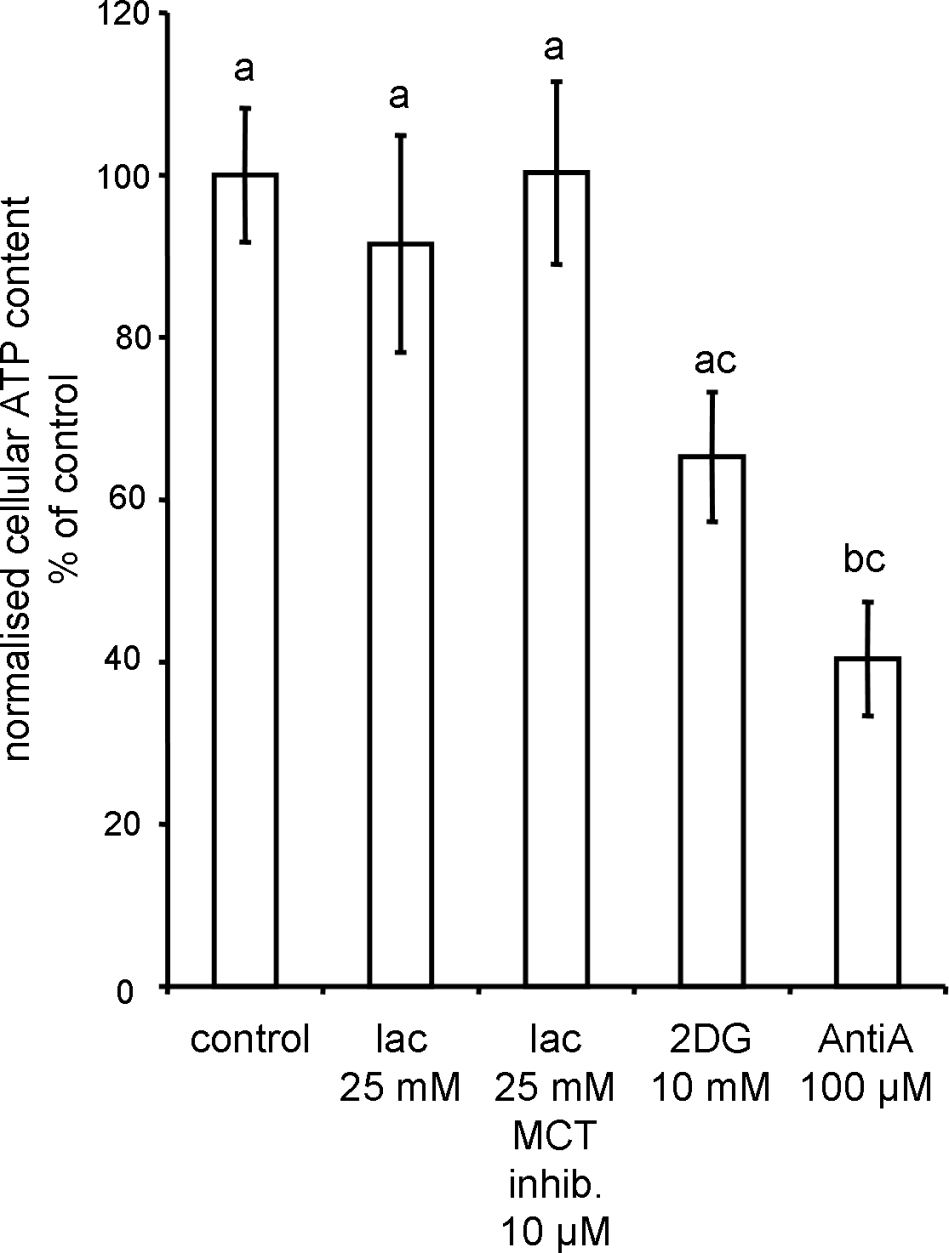
Effect of glucose and lactate on cellular ATP content in IPEC-1 cells after 1 h treatment. IPEC-1 cells were grown to confluence (7 d) and treated for 1 h with HBSS-based solutions. Supernatant was removed and ATP content was measured by a luminescence based assay. Statistical analysis was done by Kruskal-Wallis and Dunn's post-hoc test. Different superscripts indicate statistically significant (p<0.05) differences.

Lactate is a potential substrate for energy metabolism. Therefore, we have analysed the effect of lactate supplementation and inhibition of lactate transport on cellular ATP content of IPEC-1 cells by luminescence assay. After 1 h incubation with lactate and lactate in combination with ARC155858 (10 μM) the ATP content was not significantly different in comparison to untreated (1 g/L glucose) control (**Fig 6**). In contrast, application of glycolysis inhibitor 2DG or complex III inhibitor Anti A significantly decreased the ATP content relative to control measurements. We conclude that lactate supplementation did not significantly modify the overall energy status (ATP content) of IPEC-1 enterocytes *in vitro*.

## Discussion

The switch between maternal milk and solid food during weaning is a massive intervention in the microbiology and physiology of the gut of all mammals. Previous *in vivo* studies showed that early weaning of piglets results in significant decreases of glutathione-peroxidase, superoxide dismutase and total antioxidant capacities in both jejunum and colon [13]. Together with these findings a significant increase of malondialdehyde as a marker of oxidative stress was observed in jejunum but not in colon. The measured H_2_O_2_ level was significantly increased in both gut sections, but the differences were larger in jejunum in comparison to colon. A similar pattern was found when the number of *Lactobacillus spp*. was monitored. Weaning significantly reduced the number of luminal lactobacilli in jejunum and colon, however, the differences were more pronounced in jejunum. In our own investigation we found a significant reduction of lactobacilli in jejunum, but not in colon (**Fig 4B**).

In the gut the reduced glutathione (GSH) is a central player in the redox balance and ROS scavenge [3]. In experiments with weaned piglets the GSH and GSSG level was analysed in plasma as well as proximal and distal localisations in the small intestine. A significant increase in GSSG:GSH ratio was found 5 d after weaning in the tissue of the proximal small intestine indicating an oxidative stress situation. This shift was not found in the distal segment [24]. The GSSG:GSH ratio in plasma 5 d after weaning is not significantly different to day 0, however, data before weaning are not available in this study. In another investigation Robert et al. 2009 found a significant shift to GSSG in plasma of piglets as a consequence of weaning [25]. Considered together, the studies suggest a relation between shift of redox balance and weaning.

There are many sources of radicals in intestinal tissue. A broad variety of toxins and microbial pathogens occurring in feed are able to induce oxidative injury [3], for instance in response to fungal mycotoxins like deoxynivalenol (DON) [26]. The main source of ROS, especially superoxide anions, is the respiratory chain located in mitochondrial inner membrane and susceptible to toxic factors [27, 28]. Despite the findings of ROS acting as a harmful challenge to enterocytes there are also increasing numbers of publications indicating that ROS have modulating functions rather than sole toxic properties, as shown in the case of formyl peptide receptors [29]. On the other side various feed compounds and bacterial products were identified harbouring general protective or anti-oxidative capacities. Acetate found in high concentrations in the colon (up to 50 mM) reduced the Flagellin-induced Il-8 production in Caco-2 via a mechanism including tubulin-alpha acetylation [30, 31]. In weaned piglets the phosphorylation of AMP-activated protein kinase (AMPK), which serves as an energy sensor and is enhanced by peritoneal LPS challenge, was reduced to non-challenged values by a diet supplemented with 1% alpha-ketoglutarate, an intermediate of citric acid cycle [32]. In this context it is remarkable that pyruvate as a central component of the energy metabolism was found to exhibit anti-oxidative properties in a model of cultured osteoblasts challenged with H_2_O_2_ [33]. In piglets exposed to the immune-modulating and intestinal barrier compromising mycotoxin DON the effects were remarkably reduced by supplementation of glutamic acid to the feed, illuminating the role of luminal nutritive compounds in modulating physiological and pathological mechanisms in the intestinal tract [26].

Our own *in vitro* experiments, using IPEC-1 and IPEC-J2 as cell culture model, showed a reduced Anti A-mediated superoxide generation in the presence of relevant lactate (25 mM) concentrations (**Fig 1**). This effect was abrogated by the application of AR-C155858, an inhibitor of MCT1 and to a lesser extent, MCT2 [34]. Lactate transport from cytosol to extracellular compartment is important under hypoxic conditions utilizing pyruvate as an acceptor of reduction equivalents to maintain glycolytic ATP generation in the absence of oxidative respiration. However, the transport in reverse direction is also relevant e.g. in liver cells facilitating gluconeogenesis or in neurons supplied with lactic acid by astrocytes [35]. MCT1, MCT2 and MCT4 were found in the small intestine of adult pigs, but MCT2 was absent in colon [36]. The transport of lactate by MCT is H^+^ dependent [37]. The pH of the chyme in jejunum/ileum was about 6.6 however, the local pH at the surface of the epithelium may differ due to near surface growing lactobacilli. The pH of lactobacilli medium was found to be around pH 4.

Direct application of live Lactobacilli as well as the treatment of IPEC cultures with untreated filtered Lactobacilli culture supernatants massively changed the cellular F-actin structure as detected by phalloidin staining (**Fig 2F–2H)**. This effect can be mimicked by the combination of 25 mM lactate and acidic pH (pH = 4), whereas with 25 mM lactate at neutral pH the actin structure appears to be unchanged. In the present investigation we focused on the effect of lactate at physiological pH values predominantly found in the porcine small intestine and colon. However, low pH values in combination with physiological lactate lead to a remarkable loss of actin filaments. A possible mechanism responsible for this observation could be the impact on histone deacetylase activity [38]. In human colon carcinoma cell line HCT116 histone acetylation was significantly increased after 3 h application of 25 mM lactate. The authors found that lactate can act as an endogenous HDAC inhibitor. The effect of inhibition of histone deacetylase (HDAC) was also investigated with different inhibitors such as OSU-HDAC-44 [39] or HTPB [40]. Both inhibitors exhibited comparable effects on the F-actin structure. However, as we do not see any effect of lactate at neutral pH on the F-actin structure, we cannot conclude that in our experiment 25 mM lactate at neutral pH had an HDAC inhibitory effect.

As described above the GSH / GSSG system is important in cellular redox regulation and dependent on the NADPH pool [41]. The amount of NADPH is coupled to the cellular NADH content via the mitochondrial transhydrogenase [42]. The measurement of NADH autofluorescence in IPEC-1 cells as depicted in **Fig 3**shows an enhanced signal in response to lactate (25 mM) suggesting a higher amount of available NADH. The increase in NADH level was not converted into a significant increase of cellular ATP by oxidative phosphorylation (**Fig 6**) and we could hypothesize that an enhanced capacity for GSSG regeneration is available.

In the present study we have shown that extracellular lactate reduces Anti A-triggered ROS burden in enterocytes. The notion that lactate is involved in ROS modulation and scavenging mechanisms was initiated by (1) the drop of lactate and lactobacilli in the proximal segments of intestine due to weaning, which is paralleled by the change of redox profile to oxidised side after weaning [13], (2) the fact that lactobacilli as the principle source of lactate can, at least *in vitro*, adhere to enterocytes and therefore produce lactate in close proximity to them (see **Fig 5, Fig 2** and [19]), and (3) the modulating effect of lactobacilli supernatants on actin cytoskeleton, which can be mimicked by corresponding lactate concentrations and low pH. It has been shown that actin and cytoskeleton reorganisation is modulated by ROS via Nox and Rho GTPases [43].

## Conclusion

We suggest that the decrease in luminal lactic acid is, at least partially, a reason for the shift towards the oxidative side of redox balance in weaned piglets. A possible mechanism is the decreased availability of NADH necessary for the regeneration of ROS scavengers, e.g. the glutathione system.
